# The Activation Parameters of a Cold-Adapted Short Chain Dehydrogenase Are Insensitive to Enzyme Oligomerization

**DOI:** 10.1021/acs.biochem.2c00024

**Published:** 2022-03-01

**Authors:** Lucien Koenekoop, Florian van der Ent, Miha Purg, Johan Åqvist

**Affiliations:** Department of Cell & Molecular Biology, Uppsala University, Biomedical Center, SE-751 24 Uppsala, Sweden

## Abstract

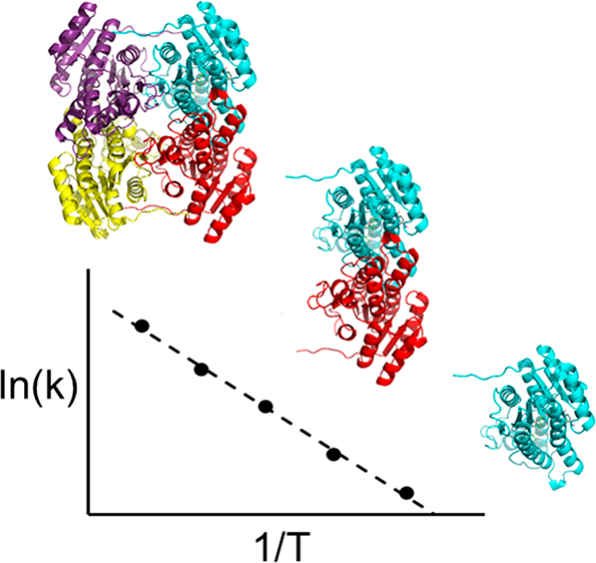

The structural principles of enzyme cold adaptation are of fundamental interest both for understanding protein evolution and for biotechnological applications. It has become clear in recent years that structural flexibility plays a major role in tuning enzyme activity at low temperatures, which is reflected by characteristic changes in the thermodynamic activation parameters for psychrophilic enzymes, compared to those of mesophilic and thermophilic ones. Hence, increased flexibility of the enzyme surface has been shown to lead to a lower enthalpy and a more negative entropy of activation, which leads to higher activity in the cold. This immediately raises the question of how enzyme oligomerization affects the temperature dependence of catalysis. Here, we address this issue by computer simulations of the catalytic reaction of a cold-adapted bacterial short chain dehydrogenase in different oligomeric states. Reaction free energy profiles are calculated at different temperatures for the tetrameric, dimeric, and monomeric states of the enzyme, and activation parameters are obtained from the corresponding computational Arrhenius plots. The results show that the activation free energy, enthalpy, and entropy are remarkably insensitive to the oligomeric state, leading to the conclusion that assembly of the subunit interfaces does not compromise cold adaptation, even though the mobilities of interfacial residues are indeed affected.

The evolutionary strategies for thermal adaptation of enzymes have attracted much attention in recent years.^1−4^ While the outcome of such adaptations is clear, namely efficient catalysis at the given environmental temperature, the structural mechanisms for achieving this are the subject of intense research. In addition to the fundamental interest in understanding structure–function relationships in proteins, this is also partly due to the biotechnological potential of rational enzyme engineering aimed at controlling the thermal characteristics of enzyme-catalyzed reactions.^5^ Understanding the structural principles of cold adaptation of natural enzymes from psychrophilic species that can maintain efficient metabolism under permanently cold conditions has been of particular interest. These enzymes are thus characterized by retaining a high catalytic activity at low temperatures, even near the freezing point of liquid water. The evolutionary pressure on enzyme activity under such conditions must differ considerably from that experienced by enzymes from thermophilic species. In the latter case, it is clear that protein stability is the key factor, where the main challenge is to resist melting at high temperatures. At low temperatures, protein stability is not really a problem, but to maintain high activity is, because chemical reaction rates decay exponentially with temperature.

A major breakthrough in the understanding of cold-adapted enzymes was made in 1973, when Somero and co-workers compared the kinetics of orthologous enzymes from ectothermic fish species living in cold water to those from warm-blooded birds and mammals.^6^ They found that the reaction kinetics of the fish enzymes was characterized by a lower enthalpy and a more negative entropy of activation than for the endothermic species. The cold-adapted enzymes were also found to generally be somewhat faster than the warm-active ones at room temperature, although the difference in activation free energy was <1 kcal/mol. The advantage with such a redistribution of the free energy components is that it is the enthalpy, and not the entropy, that causes the exponential rate decay with a decreasing temperature according to standard transition state theory
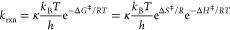
1where Δ*G*^⧧^, Δ*H*^⧧^, and Δ*S*^⧧^ are the activation free energy, enthalpy, and entropy, respectively, κ is the transmission coefficient (often assumed to be ∼1 for condensed phase reactions), and *k*_B_ and *h* are Boltzmann’s and Planck’s constants, respectively. Many subsequent studies have confirmed that this redistribution of the activation free energy components appears to be a universal feature of cold-adapted enzymes and applies to all kingdoms of life.^1−4^ Another characteristic of cold-adapted enzymes is that their melting temperature is usually lower than that of mesophilic orthologs, which, as noted above, indicates weaker evolutionary pressure on stability at their working temperatures.

Computational studies, in which the temperature dependence of Δ*G*^⧧^ is directly obtained from molecular dynamics (MD) simulations of the catalyzed reaction, have shown that flexibility of the solvent-exposed enzyme surface is a key factor in altering the balance between Δ*H*^⧧^ and −*T*Δ*S*^⧧^.^7−10^ Hence, it has been shown that mutations at surface loops where psychrophilic and mesophilic orthologs differ in mobility can alter the thermodynamic activation parameters, even though they may be far from the enzyme active site.^7−10^ Such effects have also been observed experimentally,^11,12^ and interestingly, analysis of multiple orthologous psychrophilic–mesophilic sequences typically shows conserved mutations at the enzyme surface.^7,9^ In addition, a computational experiment in which the surface of cold-adapted salmon trypsin was successively restrained, from the outside and inside, showed that this turned its psychrophilic characteristics toward those of a mesophilic ortholog.^8^ There has thus been considerable evidence accumulating that points toward the flexibility of the enzyme surface as a determinant of the temperature dependence of catalysis.

In this context, the question of whether enzyme oligomerization could be an evolutionary strategy for tuning the temperature dependence immediately arises, because parts of the surface then become engaged in intersubunit interactions. That is, if the working temperature of the enzyme is high, there could be an advantage in stabilizing its structure by oligomerization to increase its melting point (*T*_m_). On the other hand, because *T*_m_ appears to be inversely related to catalytic activity at low temperatures,^1−4^ this could possibly imply that oligomerization is actually detrimental to enzyme cold adaptation. Indeed, it has been suspected that oligomerization may enhance the stability of thermophilic and hyperthermophilic proteins. Statistical analyses of larger protein data sets did, however, not reveal any general enrichment of higher oligomers in proteins from thermophilic species.^13,14^ On the other hand, there are clearly examples of specific cases in which thermophilic and hyperthermophilic enzymes turn up as oligomers, while their mesophilic orthologs are monomeric.^15−17^ Hence, adenylate kinase from the hyperthermophilic archaeon *Sulfolobus acidocaldarius* is a trimer instead of the regular monomer,^15^ and ornithine carbamoyltransferase from *Pyrococcus furiosus* (also a hyperthermophile) shows up as a dodecamer instead of the usual trimer.^16^ A case in point is also the dihydrofolate reductase from *Thermotoga maritima* (TmDHFR). This dimeric enzyme has both its rate optimum and melting temperature (75–80 °C) increased by >30 °C compared to those of the monomeric *Escherichia coli* ortholog.^18^ Moreover, in agreement with the general trend discussed above, TmDHFR has a significantly higher activation free energy and enthalpy, accompanied by a less negative entropy, than the *E. coli* enzyme at 25 °C.^17^ Theoretical calculations on the hypothetical monomeric version of TmDHFR also predicted a much lower activation enthalpy and a more negative entropy for this variant.^17^ Hence, in this case, it seems clear that dimerization affects both the thermodynamic activation parameters and increases *T*_m_, evidently providing a strategy for thermal adaptation. Somewhat surprisingly, oligomerization has also been proposed as a mechanism for cold adaptation in the case of a β-glucosidase from an Antarctic bacterium, based on the finding that some surface regions become more flexible in its tetrameric configuration, compared to a thermophilic monomeric ortholog.^19^ In this case, however, it seems possible that cold adaptation of the enzyme is more due to sequence changes than to the actual tetramerization of the protein.

To shed further light on the role of enzyme oligomerization on thermodynamic activation parameters, in particular in relation to our earlier finding that these are directly connected to the stiffness of the protein surface,^7−10^ we address the issue here by computer simulations of a psychrophilic (*R*)-3-hydroxybutyrate dehydrogenase from *Psychrobacter arcticus* (*Pa*HBDH).^20^ This enzyme belongs to the superfamily of short chain dehydrogenases/reductases (SDRs) and catalyzes the NADH-dependent reduction of acetoacetate as well as 3-oxovalerate to (*R*)-3-hydroxybutyrate and (*R*)-3-hydroxyvalerate, respectively. The most common functional unit of the SDRs appears to be the homotetramer, but homodimers are also found in the superfamily, and even a functional monomer.^21−24^ In the case of *Pa*HBDH,^20,25^ it was found that the tetramer is present in solution and the crystal structure also showed the classical tetrameric arrangement with P- and Q-axis contacts^22,24^ between monomers. Moreover, kinetic measurements showed that with 3-oxovalerate as the substrate, the chemistry involving concerted hydride and proton transfer is rate-limiting in the temperature range of 283–318 K.^19^ Quantum mechanics/molecular mechanics (QM/MM) calculations on the 3-oxovalerate reaction further gave a detailed description of the reaction path and a good representation of the energetics.^25^ With this data in hand, we construct here an accurate empirical valence bond (EVB) model^26^ of the *Pa*HBDH reaction, which allows us to carry out extensive molecular dynamics (MD) free energy calculations of the temperature dependence of the activation free energy. This, in turn, allows us to calculate Arrhenius plots for the catalytic reaction in different oligomeric states to assess their influence on the reaction energetics.

## Methods

### MD Simulations

Models for *Pa*HBDH were based on the previously determined crystal structures in complex with NAD^+^ and acetoacetate [Protein Data Bank (PDB entry 6ZZO)] or 3-oxovalerate (PDB entry 6ZZP),^25^ where all crystallographic water molecules within 4 Å of any enzyme atom were retained. Missing residues in protein chains B and D were built using their conformation in chain A as the template. Subsequently, MolProbity^27^ was used to verify asparagine and glutamine flips and protonation states of histidine residues. All ionizable residues were assigned protonation states on the basis of their p*K*_a_ values, as predicted by PROPKA^28^ at pH 7.0, except for some residues close to the system boundary in the inactive subunits in the dimer and tetramer reaction simulations. These were taken as un-ionized to compensate for insufficient dielectric screening.^29^ The 3-oxovalerate molecule was repositioned into the active site using the Chimera program^30^ to minimize steric clashes and optimally align it for both hydride transfer from NADH and proton transfer from the catalytic acid Tyr161, as the crystal structure has several unfavorable close contacts with the substrate.

**Figure 1:**
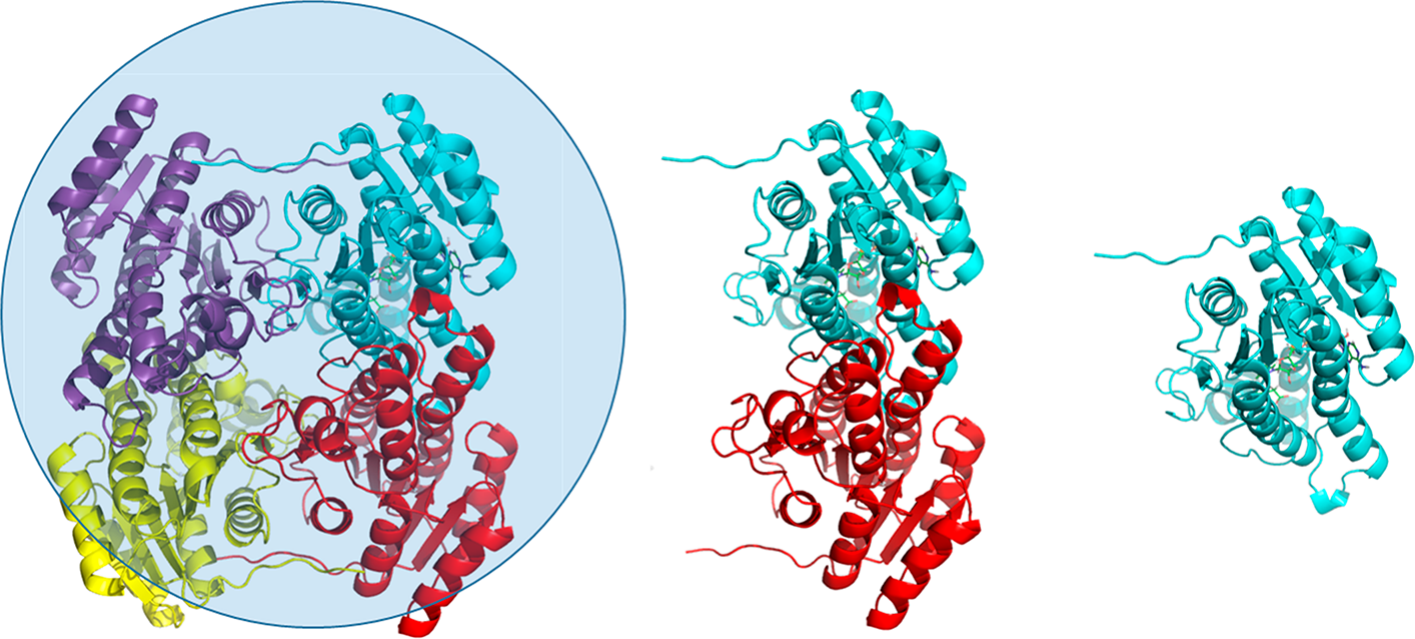
View of the three oligomeric states of the enzyme considered herein (tetramer, dimer, and monomer), with the spherical simulation system used for the tetrameric assembly indicated. The active monomer for which the MD/EVB reaction simulations are carried out is in all three cases the cyan-colored subunit.

All MD simulations were performed with the Q software package^31,32^ utilizing the OPLS-AA/M force field.^33^ Interaction parameters to describe 3-oxovalerate, (*R*)-3-hydroxyvalerate, NADH, and NAD^+^ were generated with Schrödinger’s ffld_server.^34^ The monomeric, dimeric, and tetrameric assemblies were independently solvated in spherical water droplets with diameters of 74, 84, and 90 Å, respectively, with the reactive chain and its oligomeric interfaces fully solvated (Figure 1). All atoms inside the simulation sphere were allowed to move freely, while protein atoms outside the sphere in the dimer and tetramer simulations (<3%) were tightly constrained to their initial coordinates with a force constant of 200 kcal mol^–1^ Å^–1^ and excluded from nonbonded interactions. Water molecules at the sphere boundary were subjected to radial and polarization restraints following the SCAAS model.^31,35^ The systems were partitioned into a reactive subsystem (Q atoms) and its surroundings, where the Q atoms comprised the side chain of Tyr161, the 3-oxovalerate substrate, and the nicotinamide ring and ribose moiety of NADH. All interactions of the Q atoms were calculated explicitly, while the local reaction field multipole expansion^36^ was used for other long-range electrostatic interactions, beyond a direct cutoff of 10 Å. All MD simulations employed a 1 fs time step, and a flat bottom (>2.5 Å) harmonic restraint (force constant of 10 kcal mol^–1^ Å^–1^) was applied to the distance between the donor–acceptor atom pairs in the hydride and proton transfer reactions (C···C and O···O).

### EVB Model

To calculate the Arrhenius behavior of enzyme reactions, we used the EVB method,^26^ which has been successfully employed in several earlier studies of the temperature dependence of enzyme-catalyzed reactions.^37,38^ The MD/EVB simulations of simultaneous hydride transfer from NADH and proton transfer from Tyr161 to the 3-oxovalerate substrate were based on a two-state EVB potential. The two VB states thus correspond to the diabatic reactant and product states, both described by a standard molecular mechanics force field.^33^ As described earlier, the only exception is the replacement of the Lennard-Jones potential describing the interactions between the atoms involved in bond breaking and formation by a more physical exponential repulsion *U*_rep_ = *C*_*ij*_ exp(−*a*_*ij*_*r*_*ij*_).^39,40^ This involves the hydride donor–acceptor (C···C) and proton donor–acceptor (O···O) interactions, which are both represented with *a*_*ij*_ = 4.0 Å^–1^ and *C*_*ij*_ = 2500 kcal/mol. Among the reacting groups, bonds were represented by Morse potentials (*U*_Morse_ = *D*_e_{1 – exp[−*a*(*r – r*_0_)]^2^}) obeying the relationship , where *D*_e_ is the bond dissociation energy and *k* is the harmonic force constant of the standard force field. The EVB Hamiltonian also requires the gas phase energy difference (Δα = 137.45 kcal/mol) between reactants and products and the off-diagonal coupling element (*H*_12_ = 95.92 kcal/mol) between the two VB states. These values were calibrated using the average free energy profile for the tetramer-catalyzed reaction in water, requiring that the activation and reaction free energies from earlier QM/MM calculations^25^ and experiment^20^ be exactly reproduced (at 283 K, Δ*G*^⧧^ = 16.0 kcal/mol and Δ*G*^0^ = 3.9 kcal/mol).

### Free Energy Calculations

Reaction free energy profiles were obtained using the free energy perturbation (FEP) umbrella sampling approach,^4,26^ where a mapping potential of the type *ε*_m_ = (1 – λ)ε_1_ + *λε*_2_ was used to perform biased simulations of the gradual transformation from reactants (state 1) to products (state 2), via the coupling parameter λ. This involved 51 evenly spaced λ windows between the reactant and product end-point states, with sampling for 10 ps in each window. The free energy calculations were carried out at five different temperatures between 273 and 313 K, and 100 replicate MD/EVB simulations were generated at each temperature. The simulation protocol included an initial minimization followed by a gradual heating to 293 K, with concurrent release of (10.0 kcal mol^–1^ Å^–2^) harmonic restraints on solute heavy atoms, followed by equilibration at 293 K (Figure S1). The heating/equilibration procedure involved 1.1 ns of simulation time and was applied to 100 independent replicas. For each equilibrated replica, an FEP simulation was performed at 273, 283, 293, 303, and 313 K, after additional unrestrained equilibration for 50 ps at the approximate transition state (λ = 0.5) at the target temperature. For the production stage, the mapping potential was then gradually propagated toward the reactant and product potentials (λ = 0, and λ = 1) utilizing 51 discrete λ windows, to yield a total of 51 ns of data collection for each average free energy profile at each temperature. With this protocol, the standard error of the mean (SEM) for the calculated activation free energy barriers is in all cases ≤0.14 kcal/mol. Values of the activation enthalpy and entropy were then obtained by linear regression from the corresponding Arrhenius plots of Δ*G*^⧧^/*T* versus 1/*T*. The same simulation protocol was executed for each separate oligomeric assembly.

## Results

**Figure 2:**
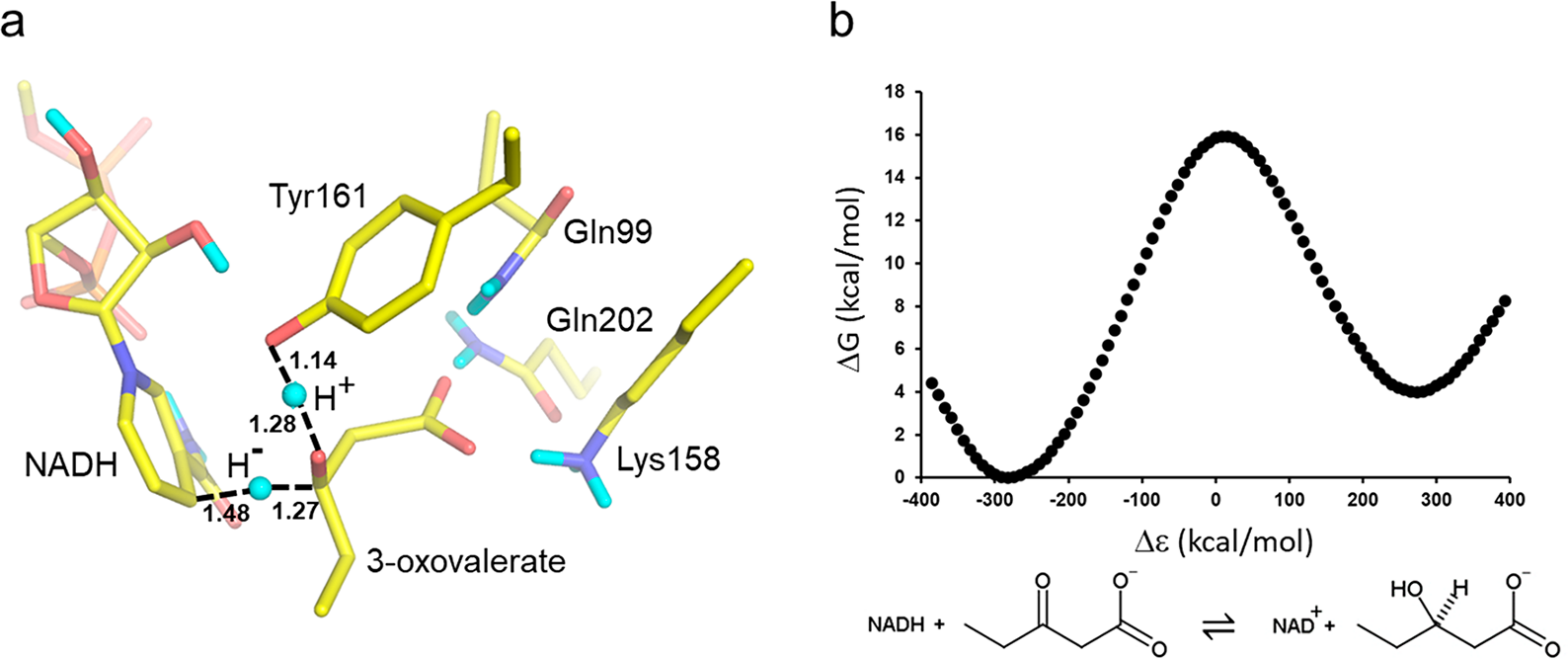
(a) View of the EVB transition state for concerted hydride and proton transfer in *Pa*HBDH, where a typical MD snapshot at the top of the free energy barrier is shown. (b) Calculated free energy profile at 10 °C for the reduction of 3-oxovalerate by *Pa*HBDH-NADH by the tetrameric form of the enzyme. Δε is the generalized reaction coordinate.^26^

An EVB model of the reaction of *Pa*HBDH with the 3-oxovalerate substrate was constructed to describe the reaction energetics using a standard force field,^33^ which allows for efficient sampling by MD simulations. Hence, the QM/MM reaction energetics at the M06-2X/ma-def2-TZVPP density functional theory (DFT) level,^25^ corrected for zero-point energy and thermal contributions, was used to calibrate the EVB model. As the QM/MM calculations involved 10 independently optimized replicas of the reaction path, we took the exponential averages of the free energies from these. The average activation free energy from this procedure was earlier found to be in near perfect agreement with the experimentally derived value (Δ*G*^⧧^ = 16.0 kcal/mol at 10 °C).^20,25^ The resulting EVB model also yields a concerted transition state for hydride and proton transfer, very similar to that from the QM/MM calculations,^25^ where the nicotinamide ring of NADH donates the hydride and Tyr161 donates the proton to the substrate (Figure 2a). The EVB parametrization involved 51 ns of MD/EVB free energy simulations at 10 °C of the reduction reaction of 3-oxovalerate by *Pa*HBDH-NADH, with the enzyme in the tetrameric state immersed in a 90 Å diameter spherical droplet and all intermolecular interfaces fully mobile (Figure 1). From these free energy simulations, the key EVB parameters, namely, the gas phase energy shift (Δα) and off-diagonal coupling element (*H*_12_),^26^ were fitted so that the resulting average free energy profile reproduces the Δ*G*^⧧^ value of 16.0 and the Δ*G*^0^ value of 3.9 kcal/mol (Figure 2b). It may be noted here that the general geometric features of the transition state can be expected to be similar among members of the SDR family, because the three-dimensional arrangement of Tyr161 and the NADH cofactor is highly conserved within the family.^21−25^

**Figure 3:**
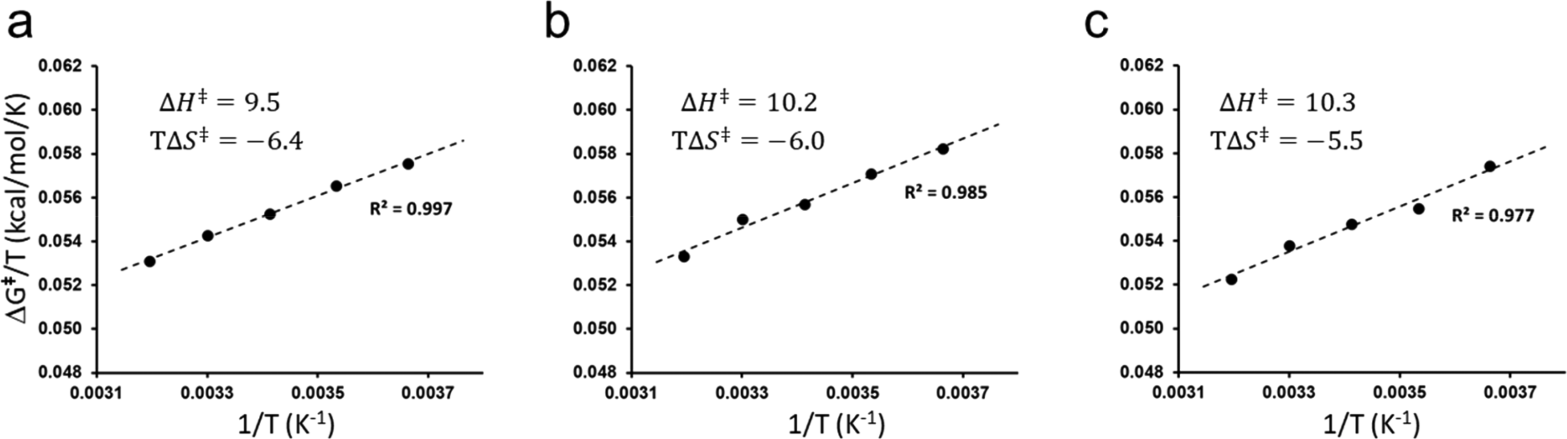
Calculated Arrhenius plots of Δ*G*^⧧^/*T* vs 1/*T* from reaction simulations at five different temperatures of (a) the tetramer, (b) the dimer, and (c) the monomer. Thermodynamic activation parameters at 283 K from the linear regressions are given, and the SEM from 100 replicate simulations for the average free energy barriers in all cases is ≤0.14 kcal/mol.

The temperature dependence of the *Pa*HBDH-catalyzed 3-oxovalerate reaction was then examined by using the parametrized EVB model and carrying out MD/EVB simulations at five different temperatures: 273, 283, 293, 303, and 313 K. At each temperature, average free energy profiles were calculated from 51 ns of data collection and the resulting Arrhenius plot of Δ*G*^⧧^/*T* versus 1/*T* was constructed to obtain the values of Δ*H*^⧧^ and Δ*S*^⧧^ (Figure 3a).^4^ Here, the standard errors of the mean (SEM) for Δ*G*^⧧^ at all temperatures are ≤0.14 kcal/mol, which shows that the free energy simulations are well converged. Remarkably, this analysis yields a Δ*H*^⧧^ value of 9.5 and a *TΔS*^⧧^ value of −6.4 kcal/mol, in near perfect agreement with the experimentally derived values (Δ*H*^⧧^ = 9.9, and *TΔS*^⧧^*=* −6.1 kcal/mol) at 283 K.^20^ It should be emphasized here that no information about the partitioning of the activation free energy into its enthalpic and entropic components enters into the EVB parametrization procedure. Hence, the fact that the experimental activation enthalpy and entropy are reproduced for this arguably complex system shows that the “curvature” of the multidimensional potential energy surface is correctly captured by the force field. That is, the *TΔS*^⧧^ term is mostly determined by the actual stiffness of the effective force field potential, which together with the constraint on the free energy barrier imposed by parametrizing the EVB model largely dictates the value of *ΔH*^⧧^. For example, if we hypothetically change the target value for *ΔG*^⧧^ in the EVB calibration to 12 kcal/mol, the resulting values of *ΔH*^⧧^ and *TΔS*^⧧^ instead become 6.0 and −5.9 kcal/mol, respectively (at 283 K). This shows that *TΔS*^⧧^ is mostly intrinsic to the force field and does not change very much when the barrier is moved up or down. The activation enthalpy, on the contrary, is more strongly correlated to the target barrier height. This reflects the fact that changing the target value for *ΔG*^⧧^ without moving Δ*G*^0^ involves a change in the off-diagonal coupling element *H*_12_ in the EVB Hamiltonian.^26^*H*_12_ is here a constant energy (enthalpy) term that lowers the free energy barrier from the intersection of the two diabatic free energy curves (with *H*_12_ = 0), corresponding to the pure EVB states of the reactants and products. This effect of *H*_12_ can easily be understood from the Marcus type of equation
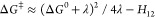
2where λ is the intrinsic reorganization free energy of the reaction.^26^

Having found that the MD/EVB simulations of the 3-oxovalerate reduction reaction catalyzed by the *Pa*HBDH tetramer yield values of *ΔH*^⧧^ (9.5 kcal/mol) and *ΔS*^⧧^ (−0.02276 kcal mol^–1^ K^–1^) that are in very good agreement with experimental data, we can now ask what the effect of oligomerization on the energetics is. We thus repeated the same calculations of reaction free energy profiles at different temperatures for the dimer and monomer with 84 and 74 Å diameter spherical systems, respectively (Figure 3b,c). To our surprise, we find that the thermodynamic activation parameters of the catalyzed reaction do not change appreciably with different oligomeric states of the enzyme. Hence, the standard SDR P-axis dimer yields a *ΔH*^⧧^ value of 10.2 and a *TΔS*^⧧^ value of −6.0 kcal/mol, and the monomer simulations give a *ΔH*^⧧^ value of 10.3 and a *TΔS*^⧧^ value of −5.5 kcal/mol, at 283 K. The corresponding free energy barriers are thus predicted to be very similar: 16.0, 16.1, and 15.8 kcal/mol for the tetramer, dimer, and monomer, respectively, with differences on the same order of magnitude as our calculated error bars for *ΔG*^⧧^. Hence, we can conclude that the thermodynamic activation parameters *ΔG*^⧧^, *ΔH*^⧧^, and *ΔS*^⧧^ are all remarkably invariant with respect to the quaternary structure of the enzyme assembly. This also shows how stable the computational Arrhenius plots resulting from MD/EVB simulations actually are.

In view of our earlier finding for cold-adapted salmon trypsin,^7,8^ that enzyme surface rigidification changes the thermodynamic activation parameters toward mesophilic characteristics, these results may seem somewhat puzzling. However, in the case of trypsin, and also cold-adapted elastase,^10^ the results showed that it was the rigidification of some specific loop regions that differ in sequence between the psychrophilic and mesophilic enzymes that caused the altered temperature dependence. Moreover, the rigidification of these loops could be achieved either by altering the amino acid sequence (mutations) or by imposing positional restraints on the loops.^8^ In the case presented here, we are comparing different oligomeric states for the same sequence and it thus appears that the packing of subunits does not involve interfaces that affect the activation parameters, which is quite interesting.

**Figure 4:**
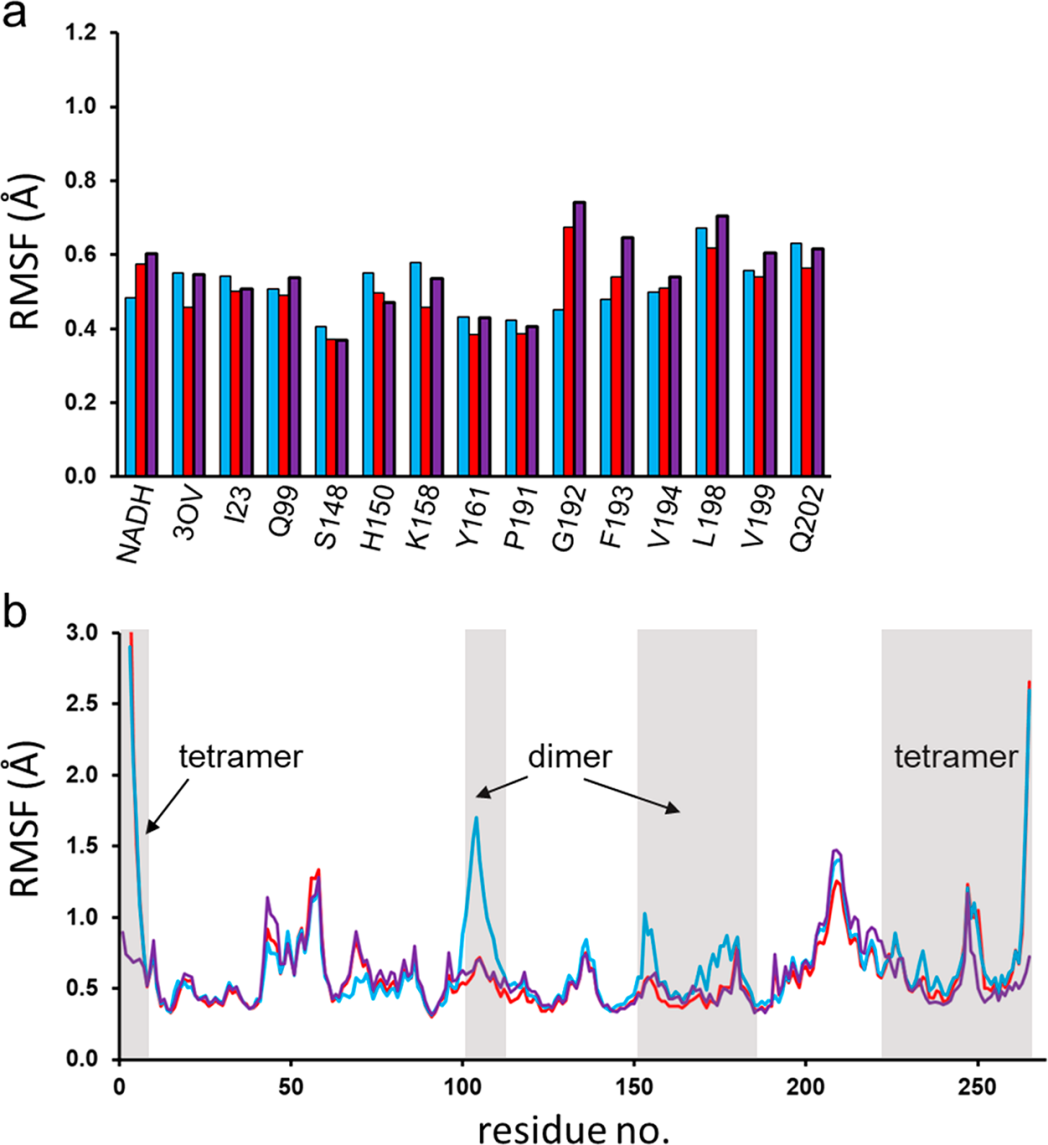
(a) Calculated average positional RMSFs per residue (heavy atoms) for the sequence region comprising the active site and substrates (NADH, 3OV), in the different oligomeric states (tetramer in purple, dimer in red, and monomer in blue). (b) Calculated average backbone RMSFs per residue along the monomeric sequence (tetramer in purple, dimer in red, and monomer in blue). Sequence regions involved in the dimer and tetramer interfaces are indicated. The RMSF calculations are done for the active monomer in all cases at 283 K.

**Figure 5:**
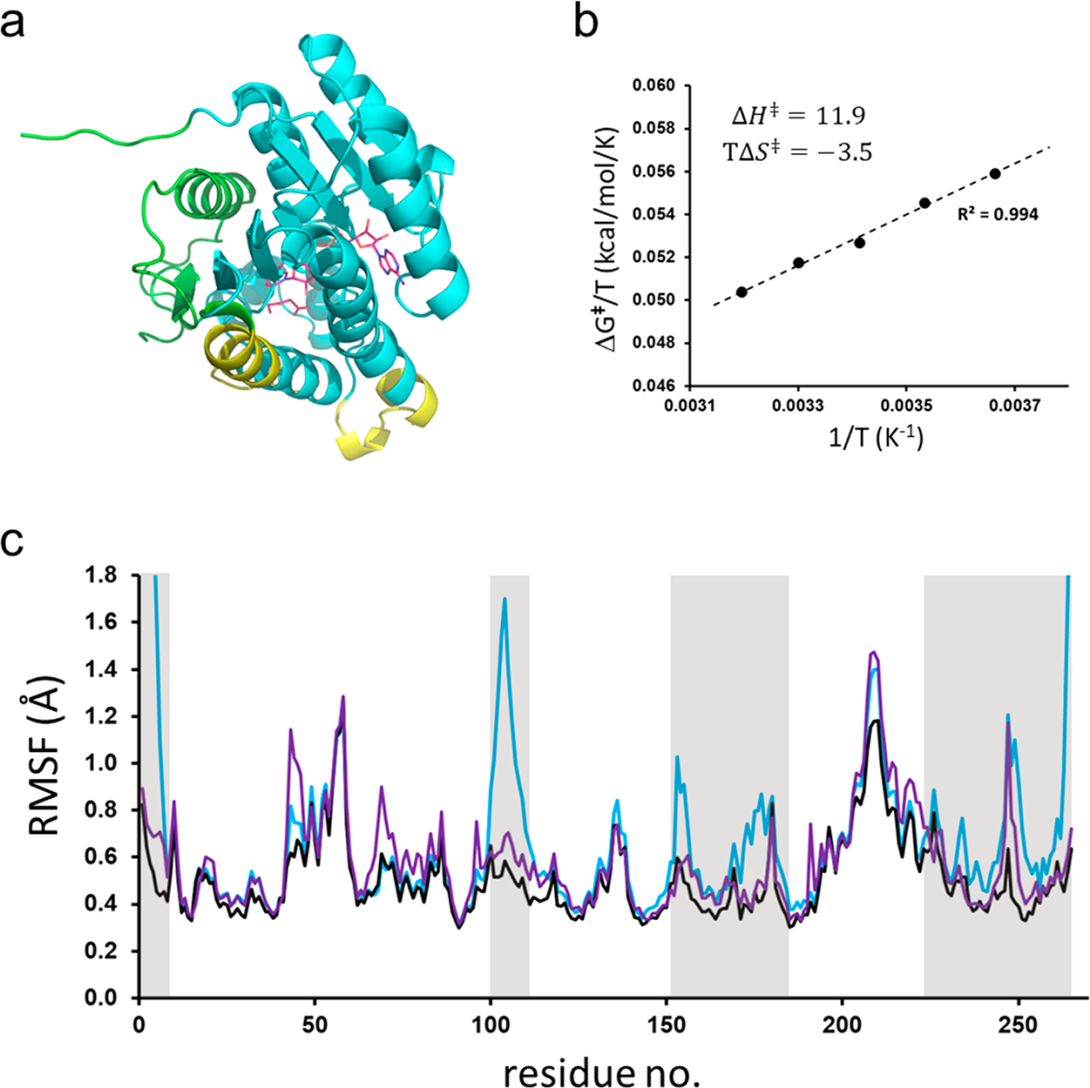
(a) View of the crystallographic monomer of *Pa*HBDH with the dimer and tetramer interfaces depicted in yellow and green, respectively. NADH and 3-oxovalerate are shown in the active site. (b) Calculated Arrhenius plot of Δ*G*^⧧^/*T* vs 1/*T* from reaction simulations at five different temperatures of the monomeric system with weak positional restraints applied to the heavy atoms of the two interfaces. Thermodynamic activation parameters from linear regression are given. (c) Calculated average backbone RMSFs per residue from MD simulations of the monomer with the dimer and tetramer interfaces restrained (black curve), compared to simulations of the free monomer (blue) and tetramer (purple). The subunit interface regions are colored gray.

To examine the relationships between protein flexibility and subunit packing more closely, we monitored how positional root-mean-square fluctuations (RMSFs) in the reactant state for different parts of *Pa*HBDH are affected by the oligomeric interactions. In agreement with earlier findings that active site mobility is more or less invariant between psychrophilic and mesophilic enzyme orthologs at a given temperature,^7,9^ the RMSFs of active site residues in *Pa*HBDH are also found to be virtually unaffected by the oligomeric state of the enzyme. Hence, considering the 13 active site residues with atoms within 5 Å of the reaction center together with NADH and the 3-oxovalerate substrate, neither the RMSFs for all their heavy atoms nor those for the protein backbone atoms only show any substantial differences at 283 K among the monomer, dimer, and tetramer (Figure 4a). As expected for highly evolved active sites, the RMSFs for this region are all small (≤0.7 Å) with little variation. Upon comparison of the average heavy atom RMSF per residue for those regions involved in forming the dimer and tetramer interfaces, the changes between the oligomeric states are as expected (Figure 4b). Hence, the RMSF pattern for the dimer interface is virtually identical for the tetramer and dimer but clearly shows increased mobility in the monomer. This pertains to the sequences of residues 102–111, 151–156, and 169–177. The first and last of these regions are at the opposite ends of the two helices in each monomer that make up the four-helix bundle dimer interface, while residues 151–156 constitute a loop region that also interacts with the bundle (Figure 5a). However, the core of the four-helix bundle has very similar and low RMSFs in all three oligomeric states, which shows that the packing of the two helices in each monomer is very stable. The average backbone RMSF plots at 303 K (Figure S2) are very similar to those at 283 K (Figure 4b) with only slightly generally increased mobilities, as expected with an increase in the temperature by 20 °C. This shows that the patterns of atomic mobilities are very robust and dictated by the secondary structure of the monomer and its packing with the other subunits in the dimer and tetramer configurations.

The tetramer interface shows a clear damping of mobility for N-terminal residues 1–6, whose ordered extended conformation observed in the crystal structure is clearly dictated by the tetramer packing. This region is thus highly mobile in the monomer and dimer simulations, while its backbone RMSFs are <1 Å in the tetrameric structure (Figure 4b). The same is true for the last three residues of the sequence that are also involved in the interface. The relatively long C-terminal tetrameric contact region between residues 224 and 262, which comprises one interfacial α-helix and one β-strand, also shows a somewhat reduced mobility in the tetramer compared to those in the dimer and monomer. However, here the effect is not very pronounced, and the average backbone RMSF only decreases from ∼0.7 to 0.5 Å, but the loop region between the α-helix and β-strand (residues 248–251) is more affected with a 35% decrease in the backbone RMSF. We also note that the sequence of residues 171–179, which involves the end of one of the α-helices in the dimeric bundle interface, is also involved in tetrameric contacts. This thus explains why the dimer and tetramer MD simulations behave similarly for this region.

To further explore the relationships between structural flexibility and thermodynamic activation parameters, we carried out additional MD/EVB simulations of the fully solvated *Pa*HBDH monomer, but with key regions of its oligomeric interfaces weakly restrained to their crystallographic positions. Harmonic restraints with a small force constant of 1 kcal mol^–1^ Å^–2^ were thus applied to the heavy atoms of residues 2–7, 103–109, 112, 154, 156, 173, 177, 178, 239, 249–255, and 264–266. Reaction free energy simulations were again performed with 100 replicas at each of the five different temperatures in the range of 273–313 K. Interestingly, this mode of reducing the surface mobility gives a clear shift in the activation parameters in the expected direction (Figure 5b). Hence, the activation enthalpy (*ΔH*^⧧^) now increases to 11.9 kcal/mol and the entropy penalty (−*TΔS*^⧧^) at 283 K is decreased to 3.5 kcal/mol, which is consistent with the interfaces becoming stiffer.^8^ This would then lead to the conclusion that oligomeric packing, which evidently does not affect the activation parameters, must exert a softer effect on the protein surface than restraining atomic positions. Indeed, this is found to be the case, and as one can see from Figure 5c, the positional restraining consistently produces RMSF values slightly lower than those of the natural tetrameric assembly. This is particularly the case for the tetramer interface, while the mobility of the dimer interface is quite similar in the tetramer and restrained simulations. This would indicate that the dimer interface actually is tighter than the tetramer one, because the effects of the natural packing more closely match the restrained simulation. At the tetramer interface, it is especially the extended N-terminal region (2–7) and residues 247–250 that are considerably more damped by the restraints than by the tetrameric packing.

It is thus noteworthy that applying weak restraints to only ∼13% of the heavy atoms of the monomer (and exclusively surface atoms) is enough to shift the thermodynamic activation parameters of the catalyzed reaction and to dampen the mobility of the oligomeric interfaces more than the actual tetramerization does. The latter observation clearly indicates that the oligomeric interfaces, although generally with RMSFs of <1 Å, still retain a sufficiently high degree of “fluidity” that does not impair the cold-adapted characteristics of the enzyme. This is also supported by the fact that our calculated balance between Δ*H*^⧧^ and *T*Δ*S*^⧧^ does not change between the monomer and tetramer.

## Discussion

The notion that a change in the oligomeric state of orthologous enzymes in differently adapted species may be a mechanism for their temperature adaptation has received unambiguous support in some cases.^15−17^ However, these appear to mainly, if not only, pertain to hyperthermophilic enzymes in which protein stability is the key issue at stake. Hence, higher oligomeric states can then offer an evolutionary tractable route to increased resistance toward protein melting. Note, however, that this necessarily involves sequence changes that can alter the preferred oligomeric state. For cold-adapted enzymes, there would seem to be little advantage with higher oligomers in terms of protein stability, because their working temperature is far below the melting point. On the other hand, there may be simpler paths for the evolution to achieve cold adaptation of an already optimized enzyme by a limited number of mutations, than to break up oligomers, as illustrated, e.g., in the case of the dimeric triosephosphate isomerase.^41,42^ However, as higher stability also has been shown to come at the cost of lower enzymatic activity,^1−3^ particularly at low temperatures, the question of whether it is actually disadvantageous for psychrophilic enzymes to exist as dimers and tetramers instead of monomers arises. The rigidification of oligomeric interfaces would then presumably be associated with a higher Δ*H*^⧧^ for the catalyzed reaction, accompanied by a less negative value of Δ*S*^⧧^, as has been observed previously in several cases.^2−4,6^ However, in the case of HBDH, we find that this is not the case and our calculations show that the monomer, dimer, and tetramer have virtually identical thermodynamic activation parameters. If anything, the tetramer shows slightly more cold-adapted characteristics than the dimer and monomer. While our MD simulations, as expected, yield a higher structural flexibility for the monomer in those regions that are involved in oligomeric contacts, these regions do apparently not affect the temperature dependence of the reaction.

The situation described above appears rather different from that observed for psychrophilic (salmon) and mesophilic (bovine) trypsins. With 66% sequence identity, distinct mobility differences were seen for a specific loop (Nβ5–Nβ6), although its backbone structure is almost identical in the two enzymes.^7^ Here, a single mutation (Y97N) in the salmon enzyme both drastically reduced the mobility of the loop and altered the chemical activation parameters toward mesophilic characteristics (and vice versa for the bovine N97Y mutation). Moreover, the same effect was observed by simply restraining the backbone of the loop in the cold-adapted salmon enzyme. Applying weak positional restraints to the oligomeric interfaces in *Pa*HBDH again produces the same effect, but we find here that their mobility then becomes more damped than in the natural oligomeric assembly. Hence, the conclusion is that these interfaces are probably more mobile than would be expected and, more importantly, that they do not interfere with cold adaptation.

## Supporting Information Available

Evolution of backbone RMSD and total potential energy during equilibration of the tetramer simulation (Figure S1) and calculated backbone RMSFs per residue for the different oligomeric states at 303 K (Figure S2) (PDF)
