# Supplementary material for: The Activation Parameters of a Cold-Adapted Short Chain Dehydrogenase Are Insensitive to Enzyme Oligomerization
Source: Biochemistry. 2022 Mar 1;61(7):514–22. doi: 10.1021/acs.biochem.2c00024 (PMC8988307; doi:10.1021/acs.biochem.2c00024)
Supplement: Supplementary file 1 — bi2c00024_si_001.pdf [file bi2c00024_si_001.pdf]

Supporting Information

The Activation Parameters of a Cold-Adapted Short Chain Dehydrogenase are Insensitive to Enzyme Oligomerization

Lucien Koenekoop, Florian van der Ent, Miha Purg and Johan Åqvist*

Department of Cell & Molecular Biology, Uppsala University, Biomedical Center, SE-751 24 Uppsala, Sweden

*Corresponding author

e-mail: aqvist@xray.bmc.uu.se

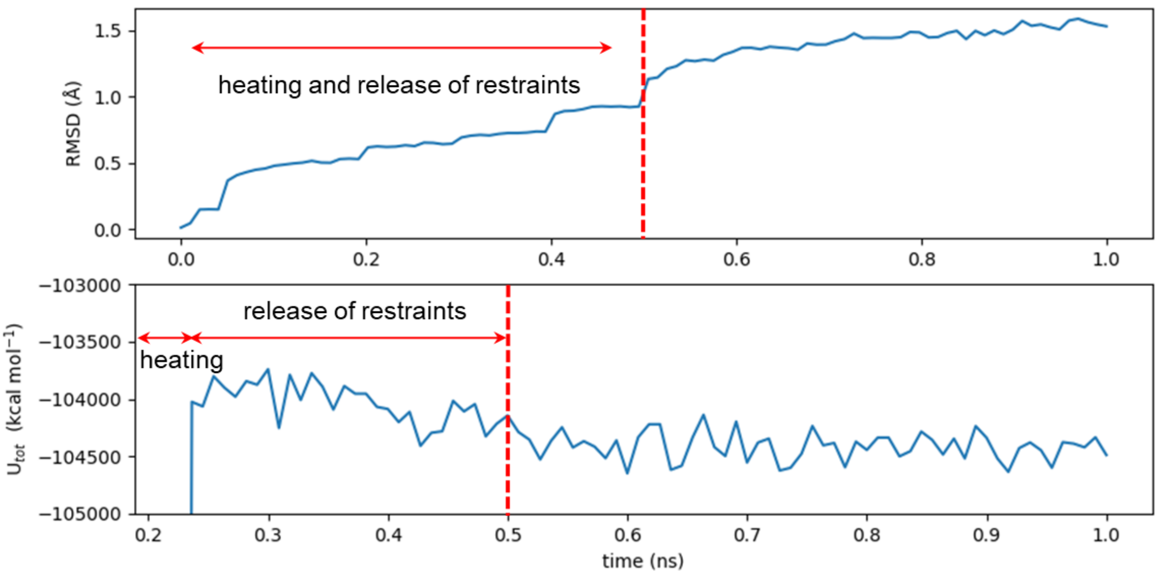

Figure S1. Evolution of the monomer backbone root-mean-square coordinate deviation from the initial structure (upper plot) and the total potential energy (lower plot), during the equilibration phase of the tetramer simulation at 293 K, where results from the first replica are shown (all replicas show a very similar behavior). The potential energy curve only includes data after the heating phase.

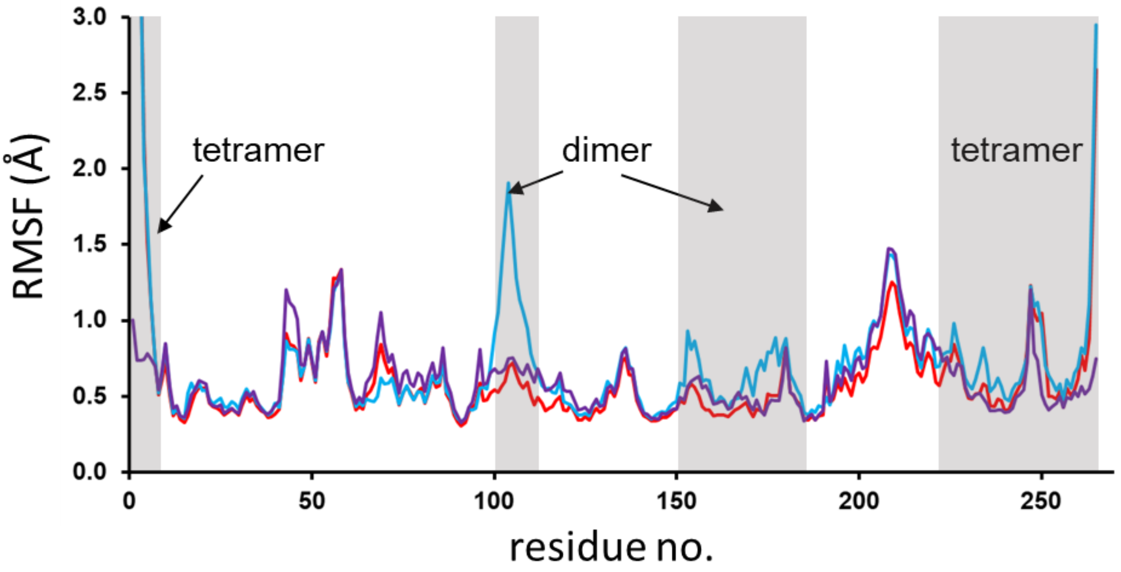

Figure S2. Calculated average backbone RMSFs per residue along the monomeric sequence at 303 K, from simulations of the different oligomeric states (tetramer – purple, dimer – red, monomer – blue). Sequence regions involved in the dimer and tetramer interfaces are indicated. The RMSF calculations are done for the active monomer in all cases.
